# Collagen proteins, thrombospondin 1 and lumican are differentially expressed across breast cancer subtypes by functional proteomics from core needle biopsy samples of Taiwanese breast cancer

**DOI:** 10.1016/j.bbrep.2025.102210

**Published:** 2025-08-31

**Authors:** Wei-Chi Ku, Nam Nhut Phan, Chih-Yi Liu, Chi-Jung Huang, Chen-Chung Liao, Yen-Chun Huang, Po-Hsin Kong, Ling-Ming Tseng, Chi-Cheng Huang

**Affiliations:** a School of Medicine, College of Medicine, Fu-Jen Catholic University, New Taipei, 242, Taiwan; b Greehey's Children Cancer Research Institute, University of Texas Health at San Antonio, San Antonio, TX, USA, 78229; c Division of Pathology, Cathay General Hospital SiJhih, New Taipei, 221, Taiwan; d Department of Medical Research, Cathay General Hospital, Taipei, 106, Taiwan; e Department of Biochemistry, National Defense Medical Center, Taipei, 114, Taiwan; f Cancer and Immunology Research Center, National Yang Ming Chiao Tung University, Taipei, 112, Taiwan; g Marker Exploration Corporation, Taipei, 112, Taiwan; h Division of Breast Surgery, Department of Surgery, Taipei Veterans General Hospital, Taipei, 112, Taiwan; i School of Medicine, College of Medicine, National Yang Ming Chiao Tung University, Taipei, 112, Taiwan; j Institute of Epidemiology and Preventive Medicine, National Taiwan University, Taipei, 100, Taiwan

**Keywords:** Breast neoplasms, Proteomics, Collagen, Thrombospondin 1, Lumican

## Abstract

**Purpose:**

This study aimed to conduct functional proteomics across breast cancer subtypes with bioinformatics analyses.

**Methods:**

Candidate proteins were identified using nanoscale liquid chromatography with tandem mass spectrometry (NanoLC-MS/MS) from core needle biopsy samples of early stage (0-III) breast cancers, followed by external validation with public domain gene-expression datasets (TCGA TARGET GTEx and TCGA BRCA).

**Results:**

Seventeen proteins demonstrated significantly differential expression and protein-protein interaction (PPI) found the strong networks including COL2A1, COL11A1, COL6A1, COL6A2, THBS1 and LUM. Public domain databases also showed that *COL2A1*, *COL11A1*, *COL6A1*, *COL6A2* and *LUM* were higher in primary/metastatic tumor than in normal tissue (one-way ANOVA, all P-values less than 0.001), and all six genes were differentially expressed across four molecular subtypes based on hormone receptor (HR) status and human epidermal growth factor receptor II (HER2) status (one-way ANOVA, all P-values less than 0.001). Disease-specific survival discrepancy was observed comparing breast cancer patients of the upper and lower quartile of the collagen family (*COL2A1*, *COL11A1*, *COL6A1*, *COL6A2*), *THBS1* and *LUM* gene expression signature (log-rank test, P = 0.06).

**Conclusion:**

Functional proteomics suggested that collagen proteins, thrombospondin 1 and lumican are differentially expressed across breast cancer subtypes.

## Highlights

• 17 functional proteins with differential expression across breast cancer subtypes.
• Strong protein-protein interaction among COL2A1, COL11A1, COL6A1, COL6A2, and THBS1.
• Collagen proteins, thrombospondin 1, and lumican are potential protein biomarkers.

## 1 Introduction

Breast cancer is one of the most common female malignancies in Taiwan and treatment outcomes have been improved tremendously due to mammography screening and availability of multi-modalities in systemic therapy [[1], [2], [3]]. Currently the arrangement of systemic adjuvant therapy for breast cancer is based on clinical and pathological prognostic and/or predictive factors such as estrogen receptor (ER), progesterone receptor (PgR) and human epidermal growth factor receptor type II (HER2) status [4]. These parameters not only determine which form of systemic therapy (i.e., chemo-/endocrine-/targeted therapy) should be prescribed but sometimes also predict therapeutic responsiveness (endocrine therapy for ER- or PR-positive and anti-HER2 targeted therapy for HER2-positive breast cancer). In addition, these factors can also be used to define breast cancer subtypes corresponding to distinct combinations of immunohistochemistry (IHC) test results [5].

The application of IHC assay for the diagnosis and treatment of breast cancer has become the standard of care as new biomarkers emerge [6]. Hormone receptor (HR) status (ER and PgR) is one of the most deterministic factors while other prognostic, but not predictive factors routinely used to assess breast cancer risk including tumor size, axillary lymph node and distant metastatic status, can be used to determine both clinical and pathological stages [7]. Morphological features such as histological classification and nuclear grade, as well as IHC proliferation marker Ki-67 also matter. Clinical applications of systemic adjuvant therapy indicate that endocrine therapies are only effective in HR-positive cases while trastuzumab (a humanized monoclonal anti-HER2 antibody) is only active in *ERBB2* amplified or HER2 over-expressed patients, and cytotoxic chemotherapy will benefit more in triple negative (TN) breast cancer, which lacks expression of ER, PR and HER2 protein [8].

Proteomics profiling of breast cancer for major molecular subtypes has been demonstrated in previous kinds of literature [[9], [10], [11]]. For instance, Gámez-Pozo A et al. demonstrated that protein expression-based probabilistic graphical models and flux balance analyses revealed that some HR-positive samples had a protein expression profile like that of TN cases with comparable clinical outcomes [9,11]. This probabilistic graphical model-based classification had prognostic value in patients with luminal A breast cancer. Another study by García-Adrián et al. demonstrated two molecular groups with differences in biological processes, including glycolysis, translation, and immune response among 125 formalin-fixed paraffin-embedded (FFPE) samples from patients with non-metastatic TN breast cancer [51]. Recent advancements in molecular technology have enabled investigators to analyze proteins in various tissue types and body fluids using mass spectrometry (MS) combined with various techniques to increase the proteome coverage [[12], [13], [14]]. It has also been advocated that genetic and proteomics profiling will improve patient categorization, optimize chemotherapy choices for low-risk patients, and potentially reveal new targeted agents for high-risk patients [52]. Proteomics profiling across breast cancer subtypes remains a domain less addressed compared to transcriptomics. Therefore, it's time to conduct proteomics study, identifying novel biomarkers, to improve our understanding of breast cancer pathogenesis and guide treatment. In this study we performed functional proteomics for a Taiwanese breast cancer cohort across distinct IHC subtypes, with extensive bioinformatics analyses for external validation.

## 2 Materials and methods

**Fig. 1:**
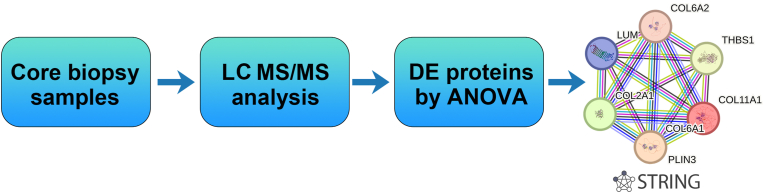
Schematic design of the study using protein-protein interaction (PPI) with functional proteomics from breast cancer core biopsy samples network. (LC MS/MS: liquid chromatography with tandem mass spectrometry, DE: differentially expressed, ANOVA: analysis of variance).

Fig. 1 shows the schema of the study.

### 2.1 Study population

We have collected pre-operative and treatment-naive core needle biopsy samples from 61 early stage (0-III) breast cancer patients from our previous study, which were stored as pathological archives between 2010 and 2014 [15]. These core needle biopsy samples were used for targeted sequencing for genetic variant analysis and MS for protein expression. Inclusion criteria were operable breast cancers with sufficient tissues for proteomics analysis. Exclusion criteria were de novo stage IV disease, paucity of specimen for peptide extraction, and incomplete IHC results. The whole study protocol and retrospective use of archived samples had been reviewed and approved by the Institutional Review Board of Taipei Veterans General Hospital; written informed consent was waived as anonymous use of archived samples was performed. Hormone receptor (HR) positivity was defined as at least 10 % of nuclei staining positive of either ER or PR with IHC assay, and patients with low ER or PR positivity (1–9 % of nuclei with positive staining) were not recruited. For HER2 status, the ASCO/CAP guidelines were adopted. IHC 3+ and IHC 2+ with fluorescence in-situ hybridization (FISH) amplification were considered HER2 overexpression. All pathological diagnoses were ascertained by a qualified pathologist (CYL). Supplementary Table 1 details TMT batch, controls and IHC subtypes and Table 1 summarizes sample counts per IHC subtype and HER2/HR status for group distribution after removing one sample with failed proteomics analysis (see below).

**Table 1:** Sample counts per immunohistochemistry assay groups.

HR		HER2		IHC	
Positive	44(73 %)	Positive	24(40 %)	HR+/HER2-(Luminal)	29(48 %)
Negative	16(27 %)	Negative	36(60 %)	HR+/HER2+(Luminal/HER2-enriched)	15(25 %)
				HR-/HER2-(Basal)	7(12 %)
				HR-/HER2+(HER2-enriched)	9(15 %)

(HR: hormone receptor, HER2: human epidermal growth factor receptor II, IHC: immunohistochemistry).

### 2.2 Peptide preparation and tandem mass tag (TMT) labeling

Proteins from the formalin-fixed paraffin-embedded (FFPE) core biopsies were extracted and trypsin digested as previously described [16]. Of the 61 breast cancer samples processed, one sample (SA25) failed to yield measurable peptide concentrations, as assessed by post-digestion bicinchoninic acid (BCA) assay (Thermo Fisher Scientific, Waltham, MA), and was excluded from downstream analyses. For TMT labeling, TMTsixplex™ Isobaric Label Reagents (Thermo Fisher Scientific) were used. To minimize batch effects, the remaining 60 samples were randomly assigned into 12 TMT 6-plex batches, each comprising five unique samples and one common reference channel (“supermix”) prepared by pooling equal amounts of peptides from all 60 samples. Sample assignments are listed in Supplementary Table 1. Two-microgram peptides from each sample was labeled and combined. We further fractionated each TMT batch into seven fractions by Pierce™ High pH Reversed-Phase Peptide Fractionation Kit (Thermo Fisher Scientific) and vacuum dried.

### 2.3 Nanoscale liquid chromatography coupled to tandem mass spectrometry (nanoLC MS/MS) and data processing

NanoLC-MS/MS analyses were performed on a nanoAcquity UPLC system (Waters, Milford, MA) connected to the Orbitrap Elite hybrid mass spectrometer (Thermo Fisher Scientific). Peptide mixtures in 0.1 % formic acid (FA) were loaded onto a C18 BEH column (75 μm ID X 25 cm) packed with 1.7-μm particles at a pore width of 130 Å (Waters, Milford, MA). The peptides were separated using a segmented gradient in 60 min from 5 % to 35 % solvent B (acetonitrile with 0.1 % FA) at a flow rate of 300 nL/min and a column temperature of 35 °C. Solvent A was 0.1 % FA in water. The MS was operated in the data-dependent mode. In brief, survey full scan MS spectra were acquired in the orbitrap (*m*/*z* 350–1600) with the resolution set to 60 K at *m*/*z* 400 and automatic gain control (AGC) target at 106. The 15 most intense ions were sequentially isolated for HCD MS/MS fragmentation and detection in the orbitrap with previously selected ions dynamically excluded for 60 s. For MS/MS, we used a resolution of 15,000, an isolation window of 2 *m*/*z* and an AGC target value of 50,000 ions, with the maximal accumulation time of 200 ms. MS/MS fragmentation was performed with normalized collision energy of 35 % and an activation time of 0.1 ms. Ions with singly and unrecognized charge state were excluded from MS/MS fragmentation. In this study, each fraction from every TMT batch was subjected to triplicate technical replicates for LC-MS/MS analysis.

Protein identification was performed by Andromeda search engine, which was incorporated in MaxQuant (v.1.6.12.0) against the SWISSPROT human sequence database (canonical + isoforms) downloaded in Oct 2019 [17,18]. The enzyme specificity was trypsin with up to two missed cleavages. Cysteine carbamidomethylation was set as a fixed modification. N-acetylation of proteins, oxidation of methionine, and formylation of lysine were set as variable modifications. The minimum peptide length was set to seven amino acids. False discovery rates (FDRs) at the peptide and protein levels were fixed at 1 %.

### 2.4 Protein quantitation and statistical analyses

**Fig. 2:**
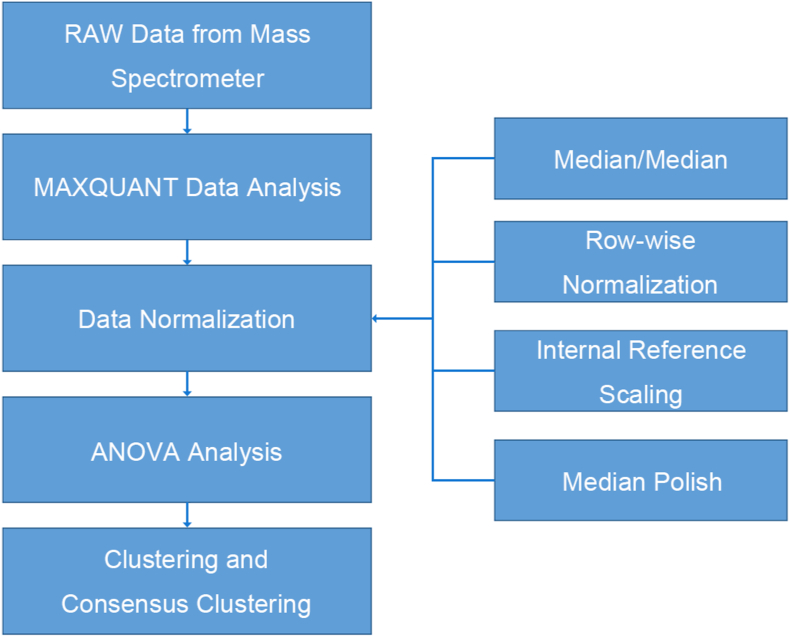
Data process flow for proteomics analysis.

Fig. 2 shows proteomics data process flow. Protein quantitation was performed by MaxQuant. First, PSM-level normalization in MaxQuant was turned on, with PIF filter >0.75 and “weighted ratio to reference channel" normalization method selected, the latter referred to the reference sample (control group, which was the mixed average of all cancerous samples) in each TMT batch [19]. For downstream data normalization and statistical analyses, the Perseus software was used [53]. Proteins quantified in at least 70 % of samples. Proteins quantified in at least 70 % of samples were retained for further analysis, and missing values were imputed using the default left-censored method in Perseus. To reduce the influence of technical variation, several normalization strategies were compared, including median-median normalization [20], row-wise normalization, internal reference scaling normalization [54], and median polish. A Separate ANOVA model was fitted for log-signal expression of each protein with all factors considered fixed effects. The F-test was based on the likelihood ratio test or type III sum of squares, with each protein expression adjusted for all others [21].

All nanoLC-MS/MS raw files and MaxQuant-generated result data have been deposited to the ProteomeXchange Consortium (http://proteomecentral.proteomexchange.org) via the PRIDE partner repository with the dataset identifier PXD038543 [22,23].

### 2.5 Protein-protein interaction

Protein-protein interaction (PPI) was deciphered using the STRING database, which is an interactive web-tool to search for the PPI network based on multiple processes and domain such as biological process, molecular function, cellular component, KEGG pathways, Reactome pathways, PFAM proteins domains, INTERPRO protein domains, SMART protein domains in the whole genome scale [24]. STRING database also provides the types of interaction between proteins such as known interactions from curated databases and experimentally determined, predicted interaction such as gene neighborhood, gene fusion, gene-co-occurrence and other text mining, co-expression, and protein homology. We used the search function: Multiple Proteins by Names/Identifiers from the STRING website (url://string-db.org/cgi/input?sessionId = baQ9KiMVeoiO&input_page_active_form = multiple_identifiers), with default settings of full STRING network, medium confidence (0.4) as required score, and medium (5 %) as FDR stringency. Only the first shell of proteins was used, as they represent the core and most enriched targets.

### 2.6 Bioinformatics analysis and gene-expression database validation

Bioinformatics analyses were carried out to validate the functional proteomics not to be spurious. Public domain database with primary/metastatic breast cancers and normal tissue and that of primary breast cancers provided opportunities of indepedent validaiton. The UCSC Xena (url://xena.ucsc.edu) was the analystical platform [25]. Supplementary Fig. 1 shows the two datasets for the comparisons between tumor and normal tissue (TCGA TARGET GTEx) as well as comparisons across four RNA-seq called PAM50 molecular subtype (TCGA BRCA) [26]. Sample types were set to “Normal Tissue”, “Primary Tumor” and “Metastatic”, while the primary site was “Breast”. For TCGA BRCA dataset, only “Primary Tumor” was selected for sample type, while PAM50Call_RNAseq was limited to “Her2”, “Basal”, “LumB” and “LumA”, mirroring the IHC subtypes from our functional proteomics study. Gene expression RNAseq values were calculated as log2(x+1) where x is the RNA-Seq by Expectation-Maximization (RSEM) value. Kaplan–Meier curves were drawn comparing the upper and the lower quartile using the log-rank test. Disease-specific survival specifically measures mortality directly attributable to breast cancer, excluding deaths from other causes.

## 3 Results

### 3.1 Differentially expressed proteins among Taiwanese breast cancers

**Fig. 3:**
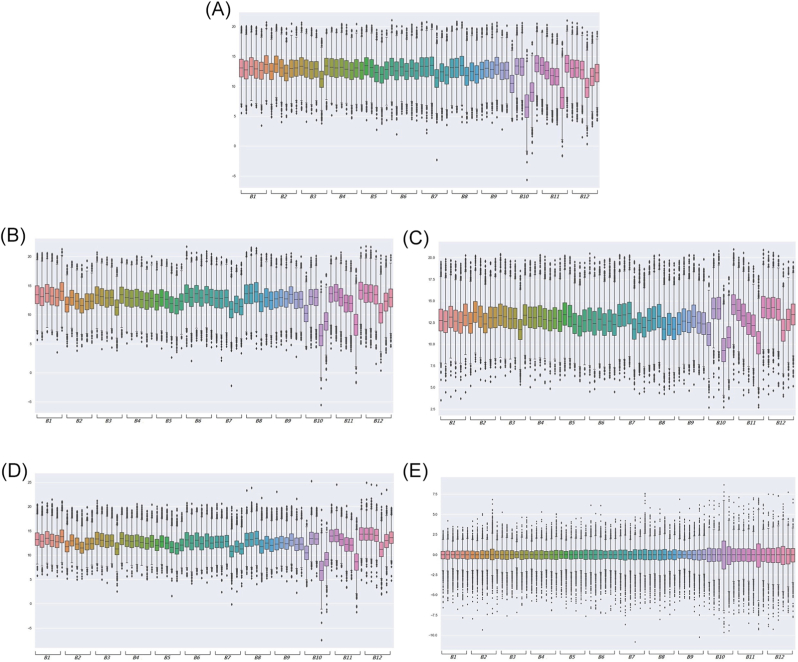
Comparison of normalization methods for TMT-based proteomics across 12 batches. Boxplots show protein intensity distributions for each TMT batch (B1–B12) across all samples: (A) Raw (unnormalized) data, (B) Median–median normalization, (C) Row-wise normalization, (D) Internal reference scaling (IRS), and (E) Median polish.

Fig. 3 summarizes the performance of several inter-batch normalization methods commonly used in TMT-based proteomics. Among them, median polish most effectively eliminated batch effects. To identify differentially expressed proteins, one-way ANOVA model was applied to each protein, using the four clinical IHC subtypes as the grouping variable. Statistical significance was defined by a permutation-based false discovery rate (FDR) < 0.05. Seventeen proteins (CAST, BCL2L13, COL6A2, PZP, LUM, COL2A1, COL6A1, COL11A1, THBS1, PLIN3, MB, TJP1, SETD7, RPL21, CLIP1, TUBB3, and TNC) were identified as significantly differentially expressed among the IHC subtypes (Table 2).

**Table 2:** Differentially expressed proteins among the four clinical IHC subtypes.

Protein	FDR	HR+/HER2+	HR+/HER2-	HR-/HER2+	HR-/HER2-
PLIN3	0.0261	0.11	0.25	−0.79	0.15
MB	0.0005	0.32	−0.02	−0.97	0.22
COL2A1	0.0245	0.28	−0.04	−0.68	−0.07
THBS1	0.0002	0.30	0.05	−1.06	0.33
COL11A1	0.0153	0.10	0.12	−0.84	0.32
COL6A1	0.0430	−0.02	0.01	−0.68	0.37
COL6A2	0.0102	0.07	0.10	−0.85	0.43
PZP	0.0484	−0.02	0.13	−0.69	0.05
CAST	0.0003	0.35	0.07	−1.06	0.22
TNC	0.0402	0.17	0.27	−0.76	−0.09
CLIP1	0.0436	0.00	0.22	−0.76	0.18
RPL21	0.0330	0.00	0.14	−0.77	0.33
LUM	0.0336	−0.05	0.20	−0.77	0.19
TJP1	0.0083	−0.12	0.21	−0.83	0.47
TUBB3	0.0170	−0.07	0.28	−0.76	−0.08
SETD7	0.0240	0.10	0.12	−0.82	0.33
BCL2L13	0.0066	0.23	0.20	−0.82	−0.26

(FDR: false discovery rate, HR: hormone receptor, HER2: human epidermal growth factor receptor II, IHC: immunohistochemistry).

### 3.2 PPI networks show important links between differentially expressed proteins

To further narrow down and investigate the interaction of differentially expressed proteins, we searched for the PPI network from the STRING database to identify potential connections.

**Fig. 4:**
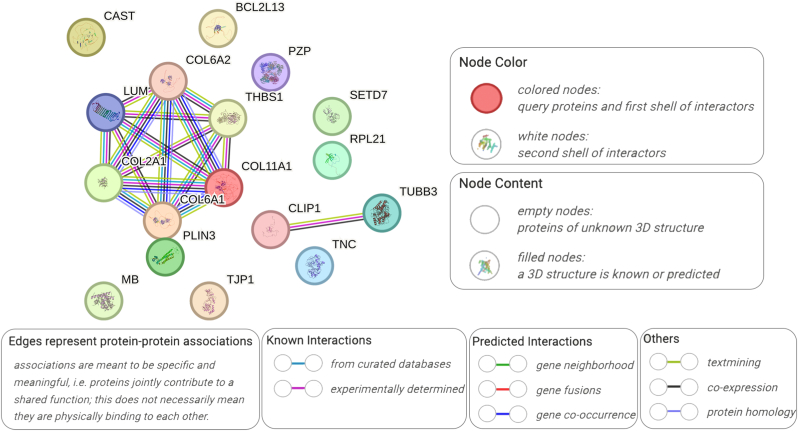
Protein-protein interaction network from 17 protein targets (CAST, BCL2L13, COL6A2, PZP, LUM, COL2A1, COL6A1, COL11A1, THBS1, PLIN3, MB, TJP1, SETD7, RPL21, CLIP1, TUBB3 and TNC).

Input variables were the 17 proteins from ANOVA (CAST, BCL2L13, COL6A2, PZP, LUM, COL2A1, COL6A1, COL11A1, THBS1, PLIN3, MB, TJP1, SETD7, RPL21, CLIP1, TUBB3 and TNC). There were highly interactive networks within these 17 proteins. The PPI network is shown in Fig. 4. We found strong networks including 6 proteins, namely collagen type II alpha 1 chain (COL2A1), collagen alpha-1(XI) chain (COL11A1), Collagen alpha-1(VI) chain (COL6A1), collagen alpha-2(VI) chain (COL6A2), thrombospondin 1 (THBS1) and lumican (LUM), which were highly connected to each other. It shows potential co-expression of these proteins as a cluster, representing intertwined edges enriched with known/predicted interactions.

### 3.3 Bioinformatics validation of the collagen family proteins, *THBS1* and *LUM* gene expression signature

**Fig. 5:**
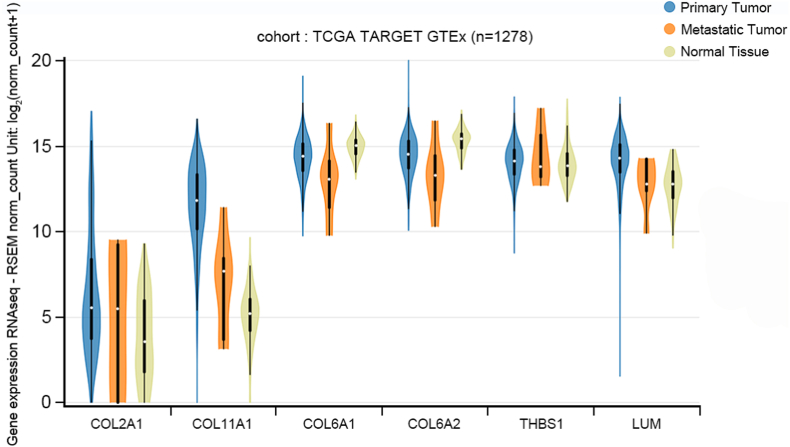
mRNA expression profiles from the TCGA TARGET GTEx (n = 1278) database. The expression of *COL2A1, COL11A1, COL6A1, COL6A2* and *LUM* tends to be higher in primary/metastatic tumor than in normal tissue.

Bioinformatics analyses with public domain database were performed, to validate the purposed signature. First, we consulted the TCGA TARGET GTEx (n = 1278), to compare the mRNA transcriptional abundance across normal tissue, primary and metastatic tumor. As shown in Fig. 5, expression of *COL2A1*, *COL11A1*, *COL6A1*, *COL6A2* and *LUM* tended to be higher in primary/metastatic tumor than in normal tissue. The results of one-way ANOVA were significant with a P-value less than 0.001 except for *THBS1* (P = 0.431).

**Fig. 6:**
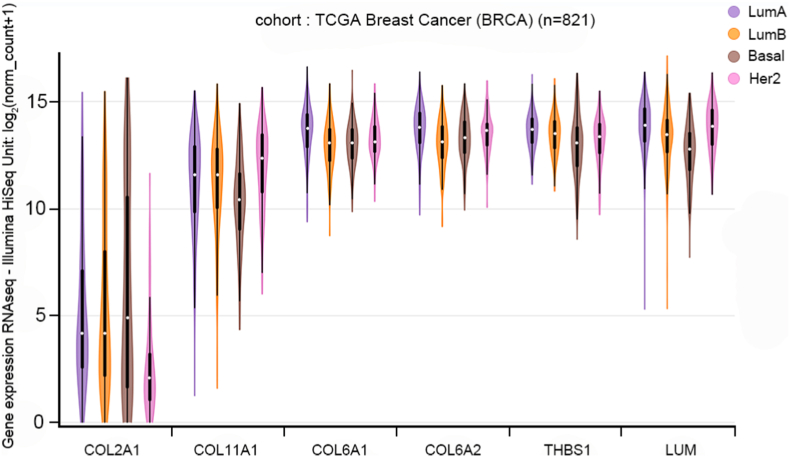
mRNA expression profiles from the TCGA BRCA (n = 821) database. *COL2A1*, *COL11A1*, *COL6A1*, *COL6A2*, *THBS1* and *LUM* were differentially expressed across four PAM50 molecular subtypes.

Second**,** the TCGA BRCA dataset (n = 821) with PAM50 subtypes called by RNAseq was used to validate the differentially expression of *COL2A1*, *COL11A1*, *COL6A1*, *COL6A2*, *THBS1* and *LUM* among four breast cancer molecular subytpes in an independent cohort (Fig. 6). One-way ANOVA tests were highly signifiant (all P-values less than 0.001), indicating the validitiy of differential expression from the purposed six genes across distinct breast cancer subtypes.

**Fig. 7:**
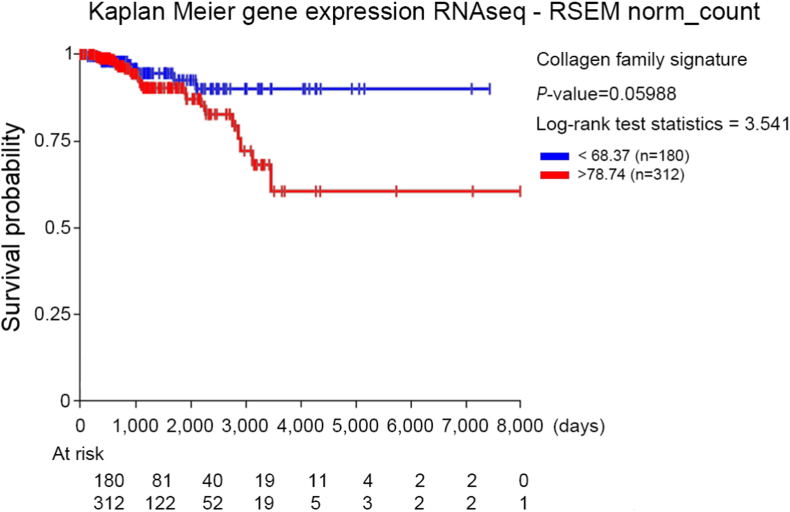
Disease-specific free survival from the TCGA BRCA cohort (n = 821). Prognostic discrepancy was observed comparing breast cancer patients of the upper and lower quartile of the collagen family, *THBS1* and *LUM* signature.

Finally, breast cancer disease-specific free survival of the upper and the lower quartile of the collagen family, *THBS1* and *LUM* signature, which was the arithmetic mean of the constitutional genes, shows a trend of survival discrepancy from the TCGA BRCA dataset (n = 821) cohort (Fig. 7, log-rank test, P = 0.06).

## 4 Discussion

In current study, we took advantage of functional proteomics from 61 Taiwanese breast cancers, 17 proteins were differentially expressed from ANOVA, and finally 6 proteins were recognized based on strong PPI networks. In addition to protein expression, public domain gene-expression database also validated the emergence of the purposed biomarkers. One merit of this study came from the methodology of LC-MS/MS for target protein identification. Most multi-gene signatures for breast cancer prognostication are mRNA (gene expression)-based, and few take protein expression as a constitutional component of a biomarker [27,28].

A couple of collagen family proteins were identified, namely COL2A1, COL11A1, COL6A1 and COL6A2. Collagen is a family of fibrous proteins that play a crucial role in the structure, strength, and elasticity of various connective tissues in the body. It is the most abundant protein in mammals, up to 30 % of the total protein mass, making up a significant portion of the extracellular matrix (ECM) [29]. There are at least 28 different types of collagens with at least one triple-helical domain reported, and type II/I are among the major types of this family. Type I collagen is found in skin, bone, tendon, ligament, and other connective tissues and provide tensile strength and is crucial for the integrity of skin and bone. On the other hand, type II collagen is the main component of cartilage, which is essential for joint flexibility and resistance to compression. Not within the major types of collagens, type VI forms beaded filaments connecting the epidermis to the dermis, constituting supramolecular assemblies [30]. Collagen XI composes three α chains, which form type V/XI hybrid collagen molecules by assembling with the α1(V) chain [31,32]. Both type II and XI belong to fibrillar collagens.

Genetic mutations in collagen α chain may result in spondyloepiphyseal dysplasia, spondyloepimetaphyseal dysplasia, achondrogenesis, hypochondrogenesis, kniest dysplasia, stickler syndrome (*COL2A1*) [33]; bethlem myopathy (*COL6A1*) and ullrich congenital muscular dystrophy (*COL6A2*) [34]; stickler syndrome, Marshall syndrome, otospondylomegaepiphyseal dysplasia and deafness (*COL11A1*) [35]. However, all these diseases are connective tissue related. Within ECM, collagens help organize and shape tissues. Collagen proteins are also capable to interact with cell surface receptors to regulate cell proliferation, differentiation, and migration, which may explain their oncological role in current study. Sun H et al. used TCGA, GTEx, and GEO databases and found that many collagen family genes were overexpressed in gastric cancers [36]. They concluded that elevated collagen genes might contribute to ECM remodeling in disease progression. Other studies supported targeting collagen proteins as their role in promoting tumor growth and creating a metastasis-prone microenvironment [37].

The importance of collagen family proteins in tumorigenesis comes from the observation that normal collagens in ECM are replaced with aberrant tumor-specific collagens during tumor remodeling process [38]. According to a pan-cancer RNAseq analysis, type I, III, IV, VI, and XVIII are among the most common up-regulated collagen types in solid tumor [37]. Plasma level of collagens, especially the COL11A1, has been reported to elevate in breast cancer [39]. *COL11A1* has been purposed as a prognostic biomarker for breast cancer as a fold change up to 5.3 was reported [40,41]. In pancreatic cancer, *COL11A1* modulates apoptotic inhibition and chemoresistance by activating the Akt/CREB/BCL-2/BAX signaling pathway [42]. High expression of *COL11A1* induces expression of Twist1 and MMP3, both of which are reported drug-resistant [43]. In ovarian cancer, *COL11A1* is associated with poor outcomes [44].

In animal study, *THBS1* inhibits angiogenesis and tumor growth but promotes metastasis to the lung, and both pro- and anti-metastasis was reported [45]. Shen J. et al. used tissue microarrays from clinical breast samples and showed that YAP/THBS1/FAK pathway modulates cell adhesion and invasiveness, forming the crosstalk between Hippo signaling pathway and focal adhesion [46]. Binding proteins to THBS1 include cathepsin G, fibronectin, MMPs, fibrinogen, collagens, TGFβ1, plasmin, neutrophil elastase, TFPI and heparin, further indicating the pivotal but transient role of THBS1 in ECM [47,48]. Intriguingly, *THBS1* can stimulate or inhibit proliferation, adhesion, motility, and survival of cells. It also deserves to notice that *THBS1* can block stem cell self-renewal [49].

The last target in current study is lumican (LUM), which belongs to proteoglycans and participates in ECM formation. Lumican is associated with epithelial-to-mesenchymal transition (EMT), cellular proliferation, migration, invasion, and adhesion [50]. In breast cancer, lumican may inhibit or even reverse the process of EMT and lumican-based therapy might be feasible. Younger age, higher tumor grade, lower ER level are associated with higher lumican expression. Lumican is also differentially expressed during tumor progression form normal tissue to invasive carcinoma.

Due to paucity of public domain proteomics repository, we took advantage of TCGA TARGET GTEx and TCGA BRCA, which provided whole transcriptome RNAseq data to provide the external and independent validation of the collagen family, THBS1 and LUM signature. We found that five out of the six purposed genes were significantly expressed in primary/metastatic tumor than in normal tissue, and all genes were differentially expressed across four molecular subtypes. It deserves notice that a trend of prognostic discrepancy was observed between the upper and lower quartile of the purposed signature, which was the arithmetic mean of the constitutional genes and was hypothesis-generating for future studies.

There are some limitations of the study. First, limited sample size from this preliminary study may hamper the generalizability of the purposed signature, and more samples enrolled in future studies may enhance the robustness of the target proteins to identify more potential therapeutic chances, albeit extensive bioinformatics validation has been performed. Second, limited sample size also renders subtype-specific biomarker discovery impossible, and again more samples enrolled in the future will augment the development of subtype-specific signature. Taken together, the study cohort (n = 61) is small for omics profiling, especially with 4 IHC subtypes and 12 TMT batches. This likely undermines statistical power and the generalizability of findings. Mechanistic links between identified proteins and functional roles in specific breast cancer subtypes are lacking as limited sample size was not powered for post-hoc analysis. Third, long-term follow-up of the study cohort was not available, and the prognostic power of the purposed algorithm will be evaluated once these data are ready, considering public domain dataset has shown prognostication of the purposed signature. Fourth, although the application of LC-MS/MS and TMT-based quantification provides functional insights, the novelty is somewhat limited by the over-reliance on transcriptomic validation, which cannot substitute for external proteomic confirmation or protein-level assays. As paucity of public domain proteomics data, we can only consult mRNA database as a surrogate for protein expression. ELISA is the standard for validation after the discovery phase of the experiments. Bioinformatic analysis can only be a complementary work. The use of RNA-seq data (TCGA TARGET GTEx and TCGA BRCA) to validate proteomics findings is methodologically weak as RNA expression does not always correlate with protein levels, especially for extracellular matrix components. Further studies using IHC or Western blotting in the same or independent cohorts could provide additional evidence of the purposed biomarkers’ relevance in breast cancer, as IHC would allow for the visualization of protein localization within the tissue context, while Western blotting could offer quantitative confirmation of the proteomics data.

It is well recognized that formalin (formaldehyde) fixation induces extensive protein cross-linking, which can complicate proteolytic digestion and mass spectrometric analysis. To address this, we adopted a heat-induced retrieval protocol with optimized digestion conditions, as reported by Wakabayashi et al., to enhance peptide recovery from cross-linked proteins [16]. In that study, the authors successfully identified over 1000 phosphopeptides from a single FFPE section using surfactant-assisted extraction and high-sensitivity LC-MS/MS, demonstrating that FFPE tissues retain proteomic integrity suitable for large-scale analysis. Additionally, lysine formylation was specified as a variable modification during protein identification to account for formalin-induced modifications.

In conclusion, our study ascertained that functional proteomics, with LC-MS/MS, is capable of target protein identification. Collagen and other ECM proteins, even those with "housekeeping" functions, can become targets in breast cancer due to their critical roles in regulating tumor progression and therapeutic resistance in current study and these targets were significantly differentially expressed across the four IHC subtypes.

## Informed consent statement

Written informed consent was waived as anonymous use of archived samples was conducted in current study (approved number: 2022-01-009C).

## Author contributions

Conceptualization, WCK and CCH; methodology NNP and CYL; formal analysis, CJH; investigation, CCL and YCH; resources, CCH; writing—original draft preparation, CCH; writing—review and editing, WCK; supervision, LMT; funding acquisition, CCH and LMT. All authors have read and agreed to the published version of the manuscript.

## Institutional Review Board statement

The whole study protocol and retrospective use of anonymized archived samples had been reviewed and approved by the Institutional Review Board (IRB) of Taipei Veterans General Hospital with the approved number: 2022-01-009C. All samples were prospectively collected in another study (protocol number: CGH-P104117, ref. 15), which was also approved by the IRB prior to the start of that study; all participants signed informed consent for further use of residual specimens as archived tissues.

## Funding

This work was supported in part by Fu-Jen Catholic University (grant number: PL-201812001-T), New Taipei City, Taiwan; 10.13039/501100011912Taipei Veterans General Hospital (grant numbers: V110E-005-3, V111E-006-3, V112E-004-3 and V112C-013), Taipei City, Taiwan; 10.13039/100008903Ministry of Health and Welfare (MOHW111-TDU-B-222-124016, MOHW112-TDU-B-222-124016, MOHW113-TDU-B-222-124016), Taipei City, Taiwan; and 10.13039/100020595National Science and Technology Council (grant number: NSTC 111-2314-B-075-063-MY3), Taipei City, Taiwan.

## Declaration of competing interest

The authors declare that they have no known competing financial interests or personal relationships that could have appeared to influence the work reported in this paper.

## Data Availability

ProteomeXchange Consortium
