# Supplementary material for: Collagen proteins, thrombospondin 1 and lumican are differentially expressed across breast cancer subtypes by functional proteomics from core needle biopsy samples of Taiwanese breast cancer
Source: Biochem Biophys Rep. 2025 Aug 31;44:102210. doi: 10.1016/j.bbrep.2025.102210 (PMC12414843; doi:10.1016/j.bbrep.2025.102210)
Supplement: Multimedia component 2 [file mmc2.docx]

**Supplementary Materials**


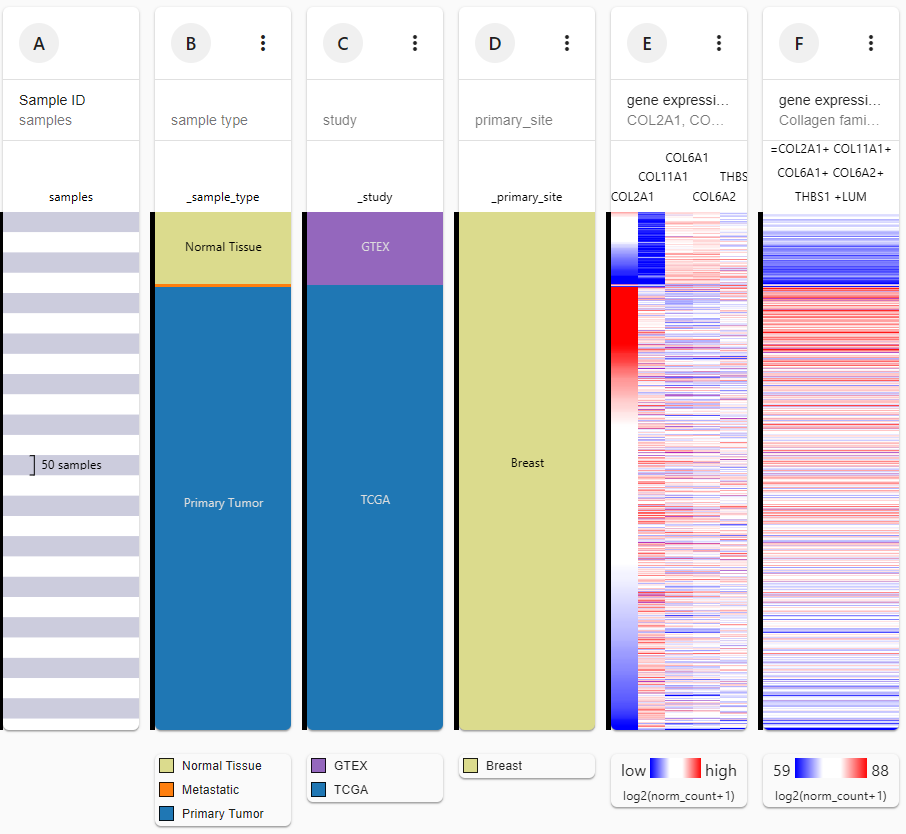


Supplementary Figure 1 (top).


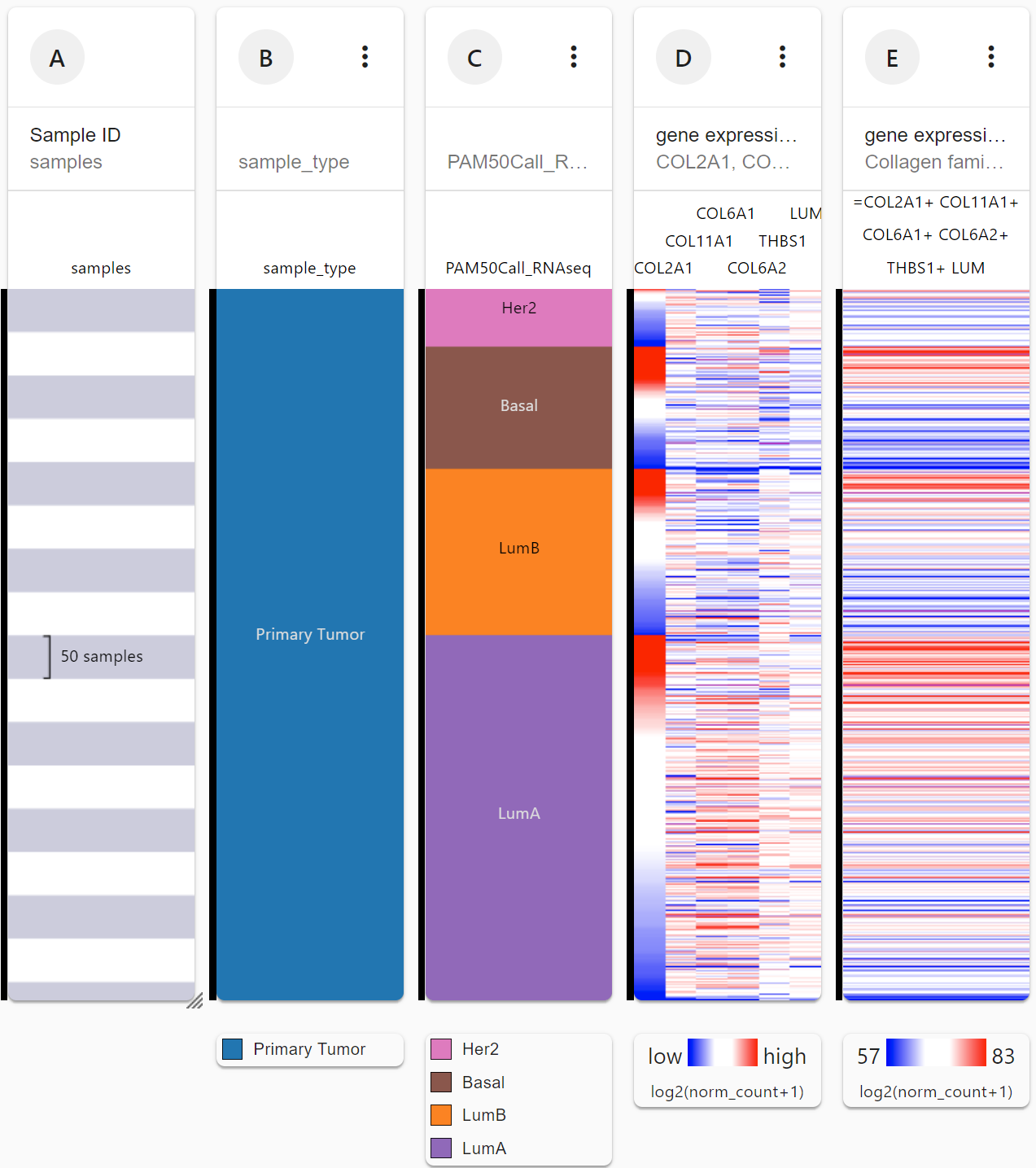


Supplementary Figure 1 (bottom).

**Supplementary Figure 1.** Public domain gene-expression databases and visual spreadsheets for the TCGA TARGET GTEx (top) and TCGA BRCA (bottom) dataset, which were operated with the UCSC Xena (url://xena.ucsc.edu) platform.
